# Biomarkers in lupus nephritis: current challenges and future perspectives

**DOI:** 10.1590/2175-8239-JBN-2025-E014en

**Published:** 2025-12-08

**Authors:** Maria Alice Sperto Ferreira Baptista

**Affiliations:** 1 Faculdade de Medicina de São José do Rio Preto, Departamento de Medicina I, Disciplina de Nefrologia, São José do Rio Preto, SP, Brazil.; 2 Hospital de Base, São José do Rio Preto, SP, Brazil.

The treatment of lupus nephritis (LN) remains one of the most challenging issues in nephrology. LN is not merely a complicating factor of systemic lupus erythematosus (SLE), but a condition that truly poses a severe threat to the kidneys and, not infrequently, to the quality of life and survival of many—often young—patients. Despite substantial progress in immunosuppressive therapies, the tools currently available to diagnose LN, monitor disease progression, and predict therapeutic response remain markedly limited.

Today, science is increasingly focusing on noninvasive biomarkers. There is a growing awareness of the need to move beyond renal biopsy, serum creatinine, and proteinuria—parameters that are critically important, but insufficient to fully capture the complexity of LN.

In a previous study of individuals treated with voclosporin in addition to mycophenolate mofetil (MMF) and corticosteroids, urinary profibrotic biomarkers (KIM-1 – kidney injury molecule 1; NGAL – neutrophil gelatinase-associated lipocalin; MCP-1 – monocyte chemotactic protein-1; and TGF-β1 – transforming growth factor beta 1) did not decrease significantly but also did not increase during treatment. This observation should be regarded as a reassuring safety signal, since these molecules reflect tubular injury and progressive fibrosis—features typically associated with the nephrotoxicity of first-generation calcineurin inhibitors. Therefore, persistently stable levels above baseline indicate that voclosporin, when combined with standard therapy (MMF and corticosteroids), did not cause additional tubular damage and confirm its favorable renal safety profile—even though these biomarkers are not sensitive indicators of therapeutic response in LN^1^.

Another review^2^ gathered evidence on noninvasive biomarkers for LN and summarized the literature on both serum and urinary markers—including cytokines, chemokines, microRNAs, cell-surface molecules, and proteins related to tubular and glomerular injury. The article emphasized that such biomarkers reflect biological processes of inflammation, injury, and renal repair, thereby providing clinical value for less invasive diagnosis and disease assessment compared to biopsy. Current limitations (heterogeneity among studies, lack of laboratory standardization, genetic and environmental factors), as well as the opportunities these tests may offer for clinical practice, were also discussed.

Similarly, Piraciaba et al.^3^ reported serum and urinary levels of suPAR (soluble urokinase plasminogen activator receptor) and VEGF (vascular endothelial growth factor) in patients with LN, in a study recently published in the *Brazilian Journal of Nephrology*. The main finding was that urinary—but not serum—suPAR correlated with glomerular hematuria and higher lupus activity (SLEDAI-2K). A key strength of this study was the demonstration that suPAR and VEGF, in contrast to NLRP3, can be reliably quantified through standardized ELISA assays, which are widely available in research and clinical laboratories. Moreover, the consistent association of urinary suPAR with hematuria and SLEDAI-2K further suggests that this parameter may serve as a surrogate marker of renal inflammation.

The authors therefore propose that urinary suPAR represents a novel potential biomarker to monitor both LN and SLE activity, being rapidly measurable in urine and clinically relevant. These are highly promising studies, and nephrologists should be encouraged to support the development of this line of research, as serial monitoring could theoretically enable early detection of renal activity changes. Patients with persistently elevated urinary suPAR may require closer surveillance or even qualify for more intensive therapeutic approaches.

As indicated by Piraciaba et al.^3^, urinary suPAR may also serve as an adjunct in risk stratification when combined with other markers such as anti-dsDNA, complement, proteinuria, NGAL, or MCP-1, thereby potentially improving diagnostic accuracy. This is an original and relevant piece of research, with particular national significance.

In this context, the convergence between data science and medicine is also of crucial importance in LN research. There are already examples of the use of artificial intelligence to analyze molecular profiles^4^, helping to predict which patients will respond best to therapy, who may be at risk of relapse, and even which treatment should be selected. The era of precision medicine and data-driven care is no longer science fiction—it is scientific fact.

Similarly, genetic profiling has been evaluated to predict therapeutic response^5^. It is highly encouraging to envision a future in which treatment can be personalized based on molecular markers—treating individuals rather than diseases. The principal aim is to reduce the empirical use of immunosuppressants and their adverse effects.

Naturally, such progress must be translated into clinical practice. Recent evidence^6,7^ emphasizes the need to incorporate this new knowledge into guidelines and diagnostic strategies. Although we remain far from the routine application of these biomarkers in daily practice, the first steps have already been taken.

The journey of biomarkers in LN is complex, but the horizon is promising. What we now require are more multicenter studies, validation across diverse populations, and, most importantly, methodological standardization. As nephrologists, we must be prepared for this change: to learn, to challenge outdated practices, and, above all, to listen to what science tells us—since our ultimate loyalty is to the patient. And it is the patients who stand to gain the most from the thoughtful and critical integration of these new tools.

## Data Availability

No new data were generated or analyzed in this study.
