# Supplementary material for: Biomarkers in lupus nephritis: current challenges and future perspectives
Source: J Bras Nefrol. 2025 Dec 8;47(4):e2025E014. doi: 10.1590/2175-8239-JBN-2025-E014en (PMC12698065; doi:10.1590/2175-8239-JBN-2025-E014en)
Supplement: Translation

# Translation: Biomarcadores na nefrite lúpica: desafios atuais e perspectivas futuras

**DOI:** 10.1590/2175-8239-JBN-2025-E014pt

**Authors:** Maria Alice Sperto Ferreira Baptista

**Affiliations:** 1 Faculdade de Medicina de São José do Rio Preto, Departamento de Medicina I, Disciplina de Nefrologia, São José do Rio Preto, SP, Brasil.; 2 Hospital de Base, São José do Rio Preto, SP, Brasil.

O tratamento da nefrite lúpica (NL) continua sendo um dos temas mais desafiadores da nefrologia. A NL não é apenas um fator complicador do lúpus eritematoso sistêmico (LES), mas uma condição que representa uma ameaça grave aos rins e, não raras vezes, à qualidade de vida e à sobrevida de muitos pacientes, frequentemente jovens. Apesar do progresso substancial nas terapias imunossupressoras, as ferramentas atualmente disponíveis para diagnosticar a NL, monitorar a progressão da doença e prever a resposta terapêutica permanecem significativamente limitadas.

Atualmente, a ciência tem se concentrado cada vez mais em biomarcadores não invasivos. Há uma crescente conscientização sobre a necessidade de ir além da biópsia renal, da creatinina sérica e da proteinúria—parâmetros que são extremamente importantes, mas insuficientes para captar totalmente a complexidade da NL.

Em um estudo anterior com indivíduos tratados com voclosporina em adição ao micofenolato de mofetila (MMF) e corticosteroides, os biomarcadores urinários pró-fibróticos (KIM-1 – molécula de lesão renal 1; NGAL – lipocalina associada à gelatinase neutrofílica; MCP-1 – proteína quimiotática de monócitos-1; e TGF-β1 – fator de crescimento transformador beta 1) não apresentaram redução significativa, mas também não aumentaram durante o tratamento. Essa observação deve ser interpretada como um sinal tranquilizador de segurança, uma vez que essas moléculas refletem lesão tubular e fibrose progressiva—características tipicamente associadas à nefrotoxicidade dos inibidores da calcineurina de primeira geração. Portanto, níveis persistentemente estáveis acima do valor basal indicam que a voclosporina, quando associada à terapia padrão (MMF e corticosteroides), não causou danos tubulares adicionais, confirmando seu perfil de segurança renal favorável—mesmo que esses biomarcadores não sejam indicadores sensíveis da resposta terapêutica na NL^1^.

Outra revisão^2^ reuniu evidências sobre biomarcadores não invasivos para NL e sintetizou a literatura referente a marcadores séricos e urinários—incluindo citocinas, quimiocinas, microRNAs, moléculas da superfície celular e proteínas relacionadas à lesão tubular e glomerular. O artigo destacou que tais biomarcadores refletem processos biológicos de inflamação, lesão e reparação renal, proporcionando, assim, valor clínico para diagnósticos e avaliações da doença menos invasivos em comparação à biópsia. Também foram discutidas as limitações atuais (heterogeneidade entre os estudos, falta de padronização laboratorial, fatores genéticos e ambientais), bem como as oportunidades que esses testes podem oferecer para a prática clínica.

De forma semelhante, Piraciaba et al.^3^ relataram os níveis séricos e urinários de suPAR (receptor ativador do plasminogênio uroquinase solúvel) e VEGF (fator de crescimento endotelial vascular) em pacientes com NL, em um estudo recentemente publicado no *Brazilian Journal of Nephrology*. O principal achado foi que o suPAR urinário — mas não o sérico—correlacionou-se com hematúria glomerular e maior atividade do lúpus (SLEDAI-2K). Um ponto forte deste estudo foi a demonstração de que o suPAR e o VEGF, em contraste com o NLRP3, podem ser quantificados de forma confiável por meio de ensaios ELISA padronizados, amplamente disponíveis em laboratórios clínicos e de pesquisa. Além disso, a associação consistente do suPAR urinário com hematúria e SLEDAI-2K sugere ainda que esse parâmetro pode atuar como um marcador substituto de inflamação renal.

Os autores propõem, portanto, que o suPAR urinário representa um novo biomarcador potencial para o monitoramento tanto da NL quanto da atividade do LES, por ser rapidamente mensurável na urina e apresentar relevância clínica. Estes são estudos altamente promissores, e os nefrologistas devem ser incentivados a apoiar o desenvolvimento desta linha de pesquisa, uma vez que o monitoramento seriado poderia, teoricamente, permitir a detecção precoce de alterações na atividade renal. Pacientes com níveis persistentemente elevados de suPAR urinário podem necessitar de vigilância mais rigorosa ou mesmo ser elegíveis para abordagens terapêuticas mais intensivas.

Conforme indicado por Piraciaba et al.^3^, o suPAR urinário também pode atuar como um marcador complementar na estratificação de risco quando combinado com outros marcadores, como anti-dsDNA, complemento, proteinúria, NGAL ou MCP-1, potencialmente aumentando a precisão diagnóstica. Trata-se de uma pesquisa original e relevante, com particular importância no contexto nacional.

Nesse contexto, a convergência entre a ciência de dados e a medicina também assume importância crucial na pesquisa sobre NL. Já existem exemplos do uso da inteligência artificial para analisar perfis moleculares^4^, auxiliando na predição de quais pacientes responderão melhor à terapia, quais podem estar em risco de recidiva e até mesmo qual tratamento deve ser selecionado. A era da medicina de precisão e dos cuidados baseados em dados deixou de ser ficção científica—tornou-se um fato científico.

De modo semelhante, o perfil genético tem sido avaliado para prever a resposta terapêutica^5^. É altamente encorajador vislumbrar um futuro no qual o tratamento possa ser personalizado com base em marcadores moleculares—tratando indivíduos em vez de doenças. O objetivo principal é reduzir o uso empírico de imunossupressores e seus efeitos adversos.

Naturalmente, esse progresso deve ser traduzido para a prática clínica. Evidências recentes^6,7^ enfatizam a necessidade de incorporar esses novos conhecimentos às diretrizes e estratégias diagnósticas. Embora ainda estejamos distantes da aplicação rotineira desses biomarcadores na prática diária, os primeiros passos já foram dados.

A jornada dos biomarcadores na NL é complexa, mas o horizonte é promissor. O que precisamos agora são mais estudos multicêntricos, validação em populações diversas e, mais importante, padronização metodológica. Como nefrologistas, devemos estar preparados para essa mudança: aprender, questionar práticas ultrapassadas e, acima de tudo, ouvir o que a ciência nos revela, uma vez que nossa lealdade máxima é para com o paciente. E são os pacientes que mais têm a ganhar com a integração cuidadosa e crítica dessas novas ferramentas.

## Disponibilidade de Dados

Nenhum dado novo foi gerado ou analisado neste estudo.
